# Sulfur-Oxidizing Bacteria Mediate Microbial Community Succession and Element Cycling in Launched Marine Sediment

**DOI:** 10.3389/fmicb.2017.00152

**Published:** 2017-02-03

**Authors:** Hideyuki Ihara, Tomoyuki Hori, Tomo Aoyagi, Mitsuru Takasaki, Yoko Katayama

**Affiliations:** ^1^United Graduate School of Agricultural Science, Tokyo University of Agriculture and TechnologyFuchu, Japan; ^2^Environmental Management Research Institute, National Institute of Advanced Industrial Science and TechnologyTsukuba, Japan; ^3^Department of Food and Environmental Sciences, Faculty of Science and Engineering, Ishinomaki Senshu UniversityIshinomaki, Japan; ^4^Institute of Agriculture, Tokyo University of Agriculture and TechnologyFuchu, Japan

**Keywords:** sulfur-oxidizing bacteria, launched marine sediment, microbial community, high-throughput sequencing, Epsilonproteobacteria

## Abstract

A large amount of marine sediment was launched on land by the Great East Japan earthquake. Here, we employed both on-site and laboratory studies on the launched marine sediment to investigate the succession of microbial communities and its effects on geochemical properties of the sediment. Twenty-two-month on-site survey showed that microbial communities at the uppermost layer (0–2 mm depth) of the sediment changed significantly with time, whereas those at the deeper layer (20–40 mm depth) remained nearly unchanged and kept anaerobic microbial communities. Nine months after the incidence, various sulfur-oxidizing bacteria (SOB) prevailed in the uppermost layer, in which afterwards diverse chemoorganotrophic bacteria predominated. Geochemical analyses indicated that the concentration of metals other than Fe was lower in the uppermost layer than that in the deeper layer. Laboratory study was carried out by incubating the sediment for 57 days, and clearly indicated the dynamic transition of microbial communities in the uppermost layer exposed to atmosphere. SOB affiliated in the class Epsilonproteobacteria rapidly proliferated and dominated at the uppermost layer during the first 3 days, after that Fe(II)-oxidizing bacteria and chemoorganotrophic bacteria were sequentially dominant. Furthermore, the concentration of sulfate ion increased and the pH decreased. Consequently, SOB may have influenced the mobilization of heavy metals in the sediment by metal-bound sulfide oxidation and/or sediment acidification. These results demonstrate that SOB initiated the dynamic shift from the anaerobic to aerobic microbial communities, thereby playing a critical role in element cycling in the marine sediment.

## Introduction

Coastal marine sediment governs the biogeochemical cycling of elements in the ocean, for instance as reservoirs of organic substances synthesized at the ocean surface (Middelburg et al., 1993) and of heavy metals (Morse and Luther, 1999). Marine sediment has diverse characteristics depending on both geographic features and human activities. On seafloor, depletion of dissolved oxygen was induced by aerobic degradation of the accumulated organic matters (Holmer and Kristensen, 1992; Cloern, 2001). Subsequently, residual organic matters are degraded by anaerobes such as sulfate-reducing bacteria (SRB). The resultant hydrogen sulfide reacts with metals including heavy metals. The reduced sulfur compounds are then preserved in the sediment (Jørgensen and Fenchel, 1974; Jørgensen, 1977). On the other hand, sulfur-oxidizing bacteria (SOB) are phylogenetically diverse and prevail in the sulfide-rich environments (Lenk et al., 2011, 2012; Dyksma et al., 2016). The accumulated reduced metal sulfides can be oxidized in the presence of oxidants by SOB, leading the heavy metal mobilization that has a critical impact on marine ecosystems. Seitaj et al. (2015) reported the seasonal change of two types of filamentous SOB affiliated to the family Beggiatoaceae in the class Gammaproteobacteria and the family Desulfobulbaceae in the class Deltaproteobacteria. However, characteristics of SOB colonizing in the sediment and their diversity are still poorly understood.

The Great East Japan Earthquake, which was the most severe earthquake recorded in Japan, occurred in the Tohoku region on 11 March 2011; the accompanying huge tsunami caused serious damage in coastal areas (Mimura et al., 2011). In addition to giving high salt stress to soil environments (Asano et al., 2013), the tsunami transported a large amount of marine sediment onto land. Up to now, the sediment has been intensively investigated to reveal its relationship with the surrounding coastal marine sediment (Tanaka et al., 2012). In addition, the risk of the sediment contaminated with heavy metals has been addressed (Kawabe et al., 2012; Tsuchiya et al., 2012; Sera et al., 2014; Nakamura et al., 2016). Concerning geochemical properties of the sediment, it has been reported that ignition loss (IL), an indicator of organic matter content, ranged from 1.2 to 16.3% and the pH range was 1.1–9.6 (Ministry of the Environment, 2011). For heavy metals, the content of As has been found to account for 1.4–32.1 mg/kg-sediment (Sera et al., 2011). However, most of the studies involved only transient data, and described the spatial and geochemical differences in the sediment. Time-course changes in the microbial and geochemical properties of the sediment after the exposure to terrestrial environments, therefore, remain to be elucidated.

Although our recent study on the launched marine sediment incubated under anaerobic conditions showed nearly unchanged microbial communities in the presence of sulfate, ferric iron and CO_2_ (Hori et al., 2014), and only amendment with nitrate facilitated the metabolic activities of anaerobic SOB in the classes Epsilonproteobacteria and Gammaproteobacteria (Aoyagi et al., 2015). Aerobic microbial activities are of considerable importance in the transformation of elements in the sediment because the surface of the sediment is always exposed to oxygen that is the highest energy-producing substrate for microbes. Nevertheless, very little is known about the structure and function of aerobic microbial communities in the sediment. In particular, SOB that use oxygen as electron donor are expected to play critical roles in the sediment because oxidation of sulfur compounds was the start of element cycle in the nitrate-supplemented incubation, while information on their physiological activities under oxic conditions is limited.

The objective of this study was to clarify microbial community succession in the launched marine sediment resulting from the exposure of the sediment to oxic conditions. To this end, we herein conducted deep sequencing of 16S rRNA genes that has provided the detailed characteristics of microbial communities (Caporaso et al., 2011; Itoh et al., 2014; Mahmoudi et al., 2015; Navarro et al., 2015). In addition to the on-site survey for 22 months, laboratory incubation of the sediment was employed to examine the short-term microbial community dynamics and the changes in geochemical properties of the sediment. Monitoring at short intervals with a special focus on the exposure to atmosphere clarified the remarkable microbial community succession mediated by SOB and their involvement in the succeeding element cycling.

## Materials and methods

### Sampling of launched marine sediment

Launched marine sediments by the Great East Japan Earthquake were collected at Higashi-matsushima, Miyagi, Japan (Table S1 and Figures S1–S3; 38°25′49″N, 141°14′39″E). Color and texture of the uppermost layer (0–2 mm depth) were reddish brown and slightly dried, whereas those of the deeper layer were black and moist. Due to the visual appearances under the environmental conditions, it was assumed that the uppermost layer was oxic and the deep layer was anoxic. The sediments were sampled from 0 to 200–300 mm depth using a spade or a core sampler, transported to the laboratory under cool, and then separated vertically into the uppermost (0–2 mm depth) and deep (20–40 mm depth) layers. These sediment samples were stored at −80°C. To select sampling date for the main examination, prior analysis of the 16S rRNA gene deep sequencing as mentioned below was performed in singlicate. Details of the prior analysis are shown in the supporting information (Figure S3). Based on the results of the analysis, we decided to use the sediments collected in December 2011, March 2012 and October 2013 for the examination of on-site changes in microbial communities and geophysical characteristics. The sediment collected in November 2012 was kept for around half a year at 4°C before conducting laboratory incubation. No significant change in microbial communities during the storage period was checked by the deep sequencing of 16S rRNA genes.

### Laboratory incubation of the sediment

The brock-state sediment was stored in a thick polyethylene bag to keep the humidity. After removing the air-exposed surface of the sediment, the inside part of the sediment remained under anoxic conditions was obtained for laboratory incubation. After thorough mixing, the sediment (approximately 200 g) was placed in a polyethylene terephthalate container (reverse truncated cone: top, 12.9 cm dia.; bottom 9 cm dia.; height 6.5 cm) to a depth of around 30 mm. These procedures were conducted in a 100-L polyvinyl fluoride bag that was filled with nitrogen gas to minimize exposure of the sediment to air. The containers with the sediment were then placed in a 27-L chamber containing water-soaked cotton to maintain 70–100% relative humidity, and incubated in the atmosphere in dark at 20–25°C for 57 days. Humidity and temperature were monitored with a data logger (Ondotori TR-72U; T&D, Nagano, Japan). Two containers were sampled destructively from on days 1, 3, 7, 14, 28, and 57. Thus, a total of 12 containers (6 dates and 2 replication) were prepared in this experiment. The 0–2 mm depth (uppermost) and 12–16 mm depth (deep) layers of the sediments were collected in duplicate from each container. Consequently, the quadruplicate samples were used for subsequent analyses. The day 0 samples were collected in quadruplicate at the beginning of the experiment.

### Geochemical analysis of the sediment

Geochemical properties measured in the uppermost layer of the on-site sediment were pH, concentrations of sulfate and metals (Na, Mg, Al, K, Ca, Fe, Cr, Cu, As, Se, Cd, and Pb). While the properties measured in the deep layer were ignition loss (IL), concentrations of ions (Na^+^, K^+^, Mg^2+^, Ca^2+^, Cl^−^, and ${\text{SO}}_{4}^{2-}$) and metals. For pH analysis, the sediment was suspended in ultra-pure water at a ratio of 1:2.5 (w/w) and then the suspension was vortexed. Following the centrifugation at 21,500 × g for 1 min at 4°C, pH of the supernatant was measured with a pH electrode (pH Meter M-12; Horiba, Kyoto, Japan). To determine IL, the sediment was dried at 100°C until the weight became constant, and then heated at 600°C for 2 h. For measurement of ion concentrations, 0.03–1.3 g of sediment was suspended with 10 mL of ultra-pure water and shaken for 30 min at 4°C. After centrifugation at 250 × g for 5 min at 4°C, the supernatant was diluted with ultra-pure water, and filtered through a cellulose acetate filter (0.2 μm pore size). The resultant samples were analyzed by an ion chromatograph (883 Basic IC Plus; Metrohm Japan Ltd., Tokyo, Japan) equipped with a Metrosep A Supp 4 column (250 × 4 mm) and a Metrosep A Supp 4/5 guard column (Metrohm Japan Ltd.) for anions, and an ion chromatograph (861 Advanced Compact IC; Metrohm Japan Ltd.) equipped with an IC YS-50 column (4.6 × 125 mm) and an IC YS-G guard column (Showa Denko, Tokyo, Japan) for cations. Detailed method for metal analysis is shown in the Supplementary Information.

During the laboratory incubation of the sediment, IL, sulfate ion concentration, pH, total carbon (TC), total nitrogen (TN), dissolved organic carbon (DOC), and dissolved nitrogen (DN) were determined. The sediment was suspended in ultra-pure water at a ratio of 1:10 (w/w) for measurement of sulfate ion concentration and at a ratio of 1:2.5 (w/w) for measurement of pH. After shaking for 30 min and centrifugation at 250 × g for 5 min at 4°C, sulfate ion concentration was measured with the ion chromatograph as described above. pH of the supernatant was measured with the pH electrode.

TC and TN of the dry sediment were measured with a carbon-nitrogen analyzer (MT-700; Yanako, Kyoto, Japan). For measurement of DOC and DN, the sediment was suspended in ultra-pure water at a ratio of 1:50 (w/w) and the solution was shaken at 4°C for 1 h. After centrifugation at 250 × g for 5 min at 4°C, the supernatant was filtered through a cellulose acetate filter (0.2 μm pore size) and DOC and DN of the supernatant were measured using a total organic carbon analyzer (TOC-VE; Shimadzu, Kyoto, Japan) connected to a total nitrogen measuring unit (TNM-1; Shimadzu).

### Extraction of DNA from the sediment, polymerase chain reaction (PCR) amplification, and deep sequencing of 16S rRNA genes

DNA was extracted from the sediment in triplicate according to the bead-beating method described by Noll et al. (2005) with some modifications: 10–20 mg of autoclaved skim milk was added to 100–500 mg of the sediment before bead beating to improve the DNA recovery, and isopropyl alcohol was used instead of ethanol for precipitation of DNA (crude extract/isopropyl alcohol/3 M sodium acetate was 10:9:1 [v/v/v]). Subsequently, RNA in the crude DNA extract was removed with RNase A (Nippon Gene, Tokyo, Japan). DNA concentration was determined spectrophotometrically (NanoDrop 2000; Thermo Scientific, Kanagawa, Japan). Eighteen and 39 DNA extracts from the on-site and incubated sediments, respectively, were utilized for the construction of deep sequencing libraries.

PCR targeting on the V4 region of 16S rRNA genes was conducted with the primer set 515F (5′-GTGCCAGCMGCCGCGGTAA-3′)/806R(5′-GGACTACHVGGGTWTCTAA-T-3′) attached to sequences for the adapter region. The reverse primer was encoded with 12-bp barcodes for multiplex sequencing (Caporaso et al., 2012). The PCR mixture included 10 μl of 5 × Q5 buffer, 1 μl of 2.5 mM dNTP, 2 μl of each of 10 pM 515F and 806R primers, 0.5 μl of DNA polymerase (Q5; NEB, Tokyo, Japan), 10 ng of template DNA, and sterile ultra-pure water for a final volume of 50 μl. PCR amplification was performed as follows: initial denaturation at 98°C for 1.5 min, and then 20 or 30 cycles consisting of denaturation at 98°C for 10 s, annealing at 55°C for 30 s, and extension at 72°C for 30 s, followed by final extension at 72°C for 2 min. The accuracy of amplification was confirmed by electrophoresis on 1.2% agarose gel. The concentration of PCR products was similar in spite of the different cycle numbers applied, which imply no or little influence of the cycle numbers on the results of the subsequent deep sequencing.

Purification of PCR products prior to deep sequencing of 16S rRNA genes was performed as described by Hori et al. (2014). The prepared DNA segments were subjected to paired-end sequencing with a 300-cycle MiSeq reagent kit (Illumina, Tokyo, Japan), and then a MiSeq sequencer (Illumina). The obtained sequences were aligned using a Burrows-Wheeler Aligner ver 0.5.9. and filtered by quality value 30 (Q30) by command lines in the software QIIME ver 1.7.0. (Caporaso et al., 2010). Chimeric sequences were removed by using the Mothur software (Schloss et al., 2009). The software QIIME was used for phylogenetic classification of operational taxonomic units (OTUs) with a cut-off value of 97% similarity. Using this software, the α-diversity indices and the weighted UniFrac distances for principal coordinate analysis (PCoA) were calculated. Some of the predominant OTUs were compared to sequences deposited in the database of the DNA Data Bank of Japan (DDBJ) using the Basic Local Alignment Search Tool (BLAST) to determine their closest relatives. The sequence data obtained in this study have been deposited in the DDBJ database under accession numbers DRA004739 and DRA004740.

### Quantitative PCR (qPCR) of the incubated sediment

To measure the number of copies of 16S rRNA genes in the sediment incubated in the laboratory, qPCR was conducted in duplicate using Premix Ex Taq II (Takara Bio Inc., Shiga, Japan) and a Thermal Cycler Dice Real Time System II (Takara Bio Inc.). The 515F/806R primer set was used and the mixture was prepared according to the manufacturer's instructions. PCR amplification was performed as follows: initial denaturation at 95°C for 30 s, and then 45 cycles consisting of 95°C for 5 s, and 61°C for 30 s. A standard curve was constructed using PCR products from the 16S rRNA gene from *Escherichia coli* with the primer set 27F (5′-AGAGTTTGATCCTGGCTCAG-3′)/1525R (5′-AAAGGAGGTGATCCAGCC-3′).

## Results

### Microbial communities in the on-site sediment

Deep sequencing of 16S rRNA genes was carried out to investigate microbial communities in the on-site sediment. The total number of sequences obtained from 18 sediment samples was around 7.2 hundred thousand, corresponding to an average of 39,775 sequences per library (Table S2). The α-diversity indices (i.e., Chao1, Shannon, and Simpson reciprocal) were calculated by using an equal number of sequences (30,789) subsampled 10 times from original libraries. These values were lower in the uppermost layer than in the deep layer, indicating that the uppermost layer had more specified and less diverse microbial communities than those in the deep layer.

**Figure 1:**
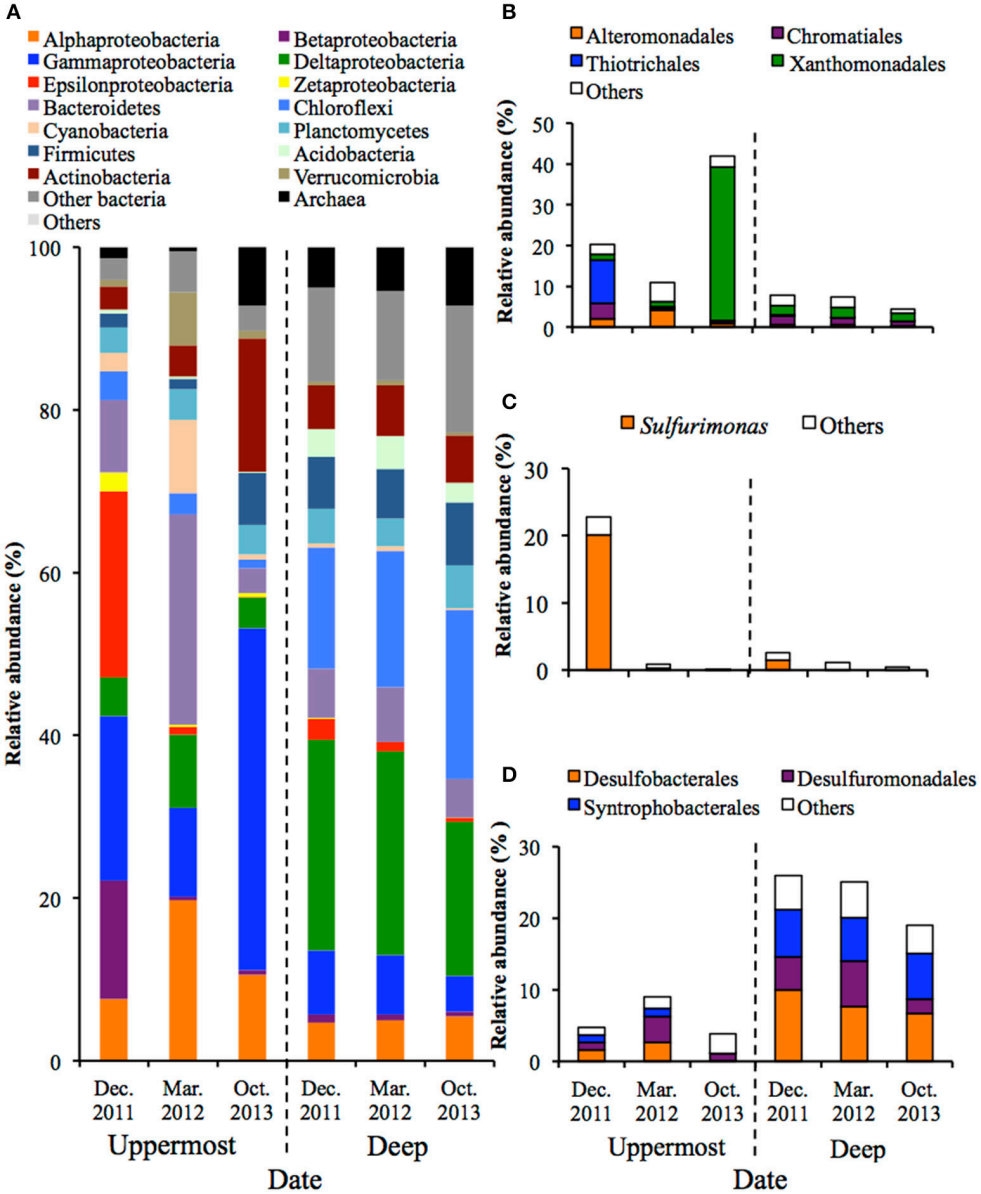
**Microbial community structures in the uppermost (0–2 mm depth) and deep (20–40 mm depth) layers of the on-site sediments based on the 16S rRNA gene analysis (***n*** = 3)**. The bars indicate average values of three replications. Sediment samples were collected in December 2011, March 2012 and October 2013. **(A)** Microbial communities are categorized by phylum except for Proteobacteria that is shown by class. The fraction of the dominant phylotypes (>3% of each library) in the classes Gammproteobacteria **(B)**, Epsilonproteobacteria **(C)**, and Deltaproteobacteria **(D)** are shown in the histograms.

PCoA illustrated that microbial communities in the uppermost layers of the sediments changed drastically during the monitored period (Figure S4). Phylogenetic information of the entire structures and predominant OTUs is shown in Figure 1 and Table S3. Figure 1 shows that the phylum Proteobacteria dominated in both the uppermost and deep layers, which accounted for 42.0–72.4% and 29.9–42.2% of the relative abundance, respectively. The class Gammaproteobacteria was predominant in the uppermost layer (relative abundances: 10.9–42.0%), and analysis at the major order showed the clear bacterial succession depending on the sampling date. More specifically, the order Thiotrichales was predominant in December 2011 (10.5%), whereas the order Xanthomonadales became dominant in October 2013 (37.7%) (Figure 1B). It is worth noting that the dominant constituent of Thiotrichales detected in the sediment was only SOB belonging in the genus *Thiomicrospira* (Table S3). With respect to other SOB, the genus *Sulfurimonas* in the class Epsilonproteobacteria was dominant in December 2011 (Figure 1C). Also, *Pandoraea thiooxydans* (OTU 1598) in the class Betaproteobacteria accounted for 12.4% in the same time (Table S3). These results indicate that SOB was present and may have performed sulfur oxidation in the uppermost layer of the sediment. In October 2013, chemoorganotrophic bacteria in the order Xanthomonadales and the phylum Actinobacteria became dominant in the uppermost layer (Figures 1A,B). Organic compounds including carbon products of SOB would serve as substrate for the chemoorganotrophs.

In contrast, PCoA and phylogenetic analysis showed microbial communities in the deep layers remained nearly unchanged over 22 months (Figure S4 and Figure 1). The class Deltaproteobacteria was dominant (19.0–25.9%) and mainly comprised the three orders (i.e., Desulfobacterales, Desulfuromonadales and Syntrophobacterales) (Figure 1D). These taxa are known to include obligate anaerobic SRB, implying that the sulfate reduction was retained under the presumably anoxic conditions of the deep layer, which is in accordance with findings obtained in our previous studies (Hori et al., 2014; Aoyagi et al., 2015).

### Geochemical properties of the on-site sediment

Geochemical analyses were conducted to characterize chemical components of the sediment and their time-dependent changes under oxic conditions. IL and ion concentrations of the deep layer were consistently high, indicating that the sediment exhibited the high accumulation of organic matters and the salinity, and these levels were kept for at least the period monitored around 22 months (Table 1). The most abundant metal in the deep layer was Al, followed by Fe (Table 2). Metals, such as Na, Mg, K, and Ca that are common in natural environments, were also found in the deep layer. Concentrations of metals other than Fe were apparently lower in the uppermost layer than those in the deep layer. While pH of the uppermost layer in March 2012 was neutral (pH 7.1), that in December 2011 was acidic (pH 4.3) (Table S1). The acidification of the uppermost layer may have facilitated the metal mobilization, resulting in the low concentration of the metals. High concentrations of sulfate in October 2013 implied that sulfur oxidation occurred in the uppermost layer. Although concentrations of heavy metals such as Cu, As, Cd and Pb were high in the sediment compared to those in soils (Iimura, 1981), their concentrations were sufficiently below the environmental standard values in the Soil Contamination Countermeasure Act of Japan (http://www.env.go.jp/en/water/soil/contami_cm.pdf).

**Table 1:** **Changes of IL and ion concentrations in the on-site sediment^a^ (***n*** = 3)**.

**Sampling date**	**IL (%)**	**Ion concentration (mg/kg dry sediment)**					
		**Na^+^**	**K^+^**	**Mg^2+^**	**Ca^2+^**	**Cl^−^**	**${\text{SO}}_{4}^{2-}$**
Dec. 2011	11.7 ± 1.5	8232 ± 2074	1780 ± 933	1963 ± 786	4000 ± 2545	12,570 ± 1504	5054 ± 683
Mar. 2012	11.4 ± 0.9	11,743 ± 4195	1008 ± 291	1473 ± 84	2391 ± 1205	16,081 ± 6564	4752 ± 1324
Oct. 2013	10.2 ± 0.3	4473 ± 1264	884 ± 113	935 ± 120	2447 ± 1094	5461 ± 1418	6827 ± 1882

a *The deep (20–40 mm depth) layer sediments were used for the analysis. The symbol “±” means the standard deviation of three replications. There was the significant difference in Cl^−^ between the sediment in March 2012 and October 2013 (p < 0.05)*.

**Table 2:** **Changes of metal concentrations in the on-site sediment**.

**Layer**	**Sampling date**	**Metal concentration (mg/kg dry sediment)**					
		**Na**	**Mg**	**Al**	**K**	**Ca**	**Fe**
Uppermost^a^	Mar. 2012	15,650	10,626	60,561	5642	8173	148,088
Deep^b^	Dec. 2011	16,673 ± 808	17,045 ± 398	113,292 ± 4022	8912 ± 182	10,242 ± 38	72,139 ± 3467
	Mar. 2012	20,477 ± 1426	18,130 ± 710	115,629 ± 2808	9083 ± 118	9600 ± 343	73,939 ± 1514
	Oct. 2013	12,035 ± 1367	15,440 ± 1649	102,379 ± 11,373	8456 ± 722	9546 ± 865	69,636 ± 5783
		**Cr**	**Cu**	**As**	**Se**	**Cd**	**Pb**
Uppermost^a^	Mar. 2012	30.7	44	32.5	1.8	0.19	30.5
Deep^b^	Dec. 2011	57.2 ± 1.7	123 ± 0.3	47.8 ± 1.7	2.5 ± 0.1	1.1 ± 0.0	52.1 ± 0.4
	Mar. 2012	57.6 ± 1.6	120 ± 4.1	47.7 ± 1.4	3.0 ± 0.1	1.1 ± 0.1	50.1 ± 0.6
	Oct. 2013	53.1 ± 5.2	118 ± 11.1	43 ± 2.8	2.8 ± 0.5	1.1 ± 0.1	53.3 ± 0.4

a *Sediment sample was collected at 0–2 mm depth from the surface. The measurement was conducted in singlicate because the quantity of obtained sample was small*.

b *Sediment samples were collected at 20–40 mm depth from the surface (n = 2) and the symbol “±” means the variation between two replications*.

### Succession of the sediment microbial communities during laboratory incubation

The on-site sediment was influenced by various environmental factors such as air exposure, insolation and rainfall, and it makes difficult to evaluate the relationship between the succession of microbial communities and the surrounding environment conditions. Thus, laboratory incubation was conducted to monitor the microbial responses to environmental changes more concretely under controlled conditions. We focused on the exposure to atmosphere because the microbial metabolism, such as chemoorganotrophic and chemolithotrophic transformation, are highly affected by redox conditions.

**Figure 2:**
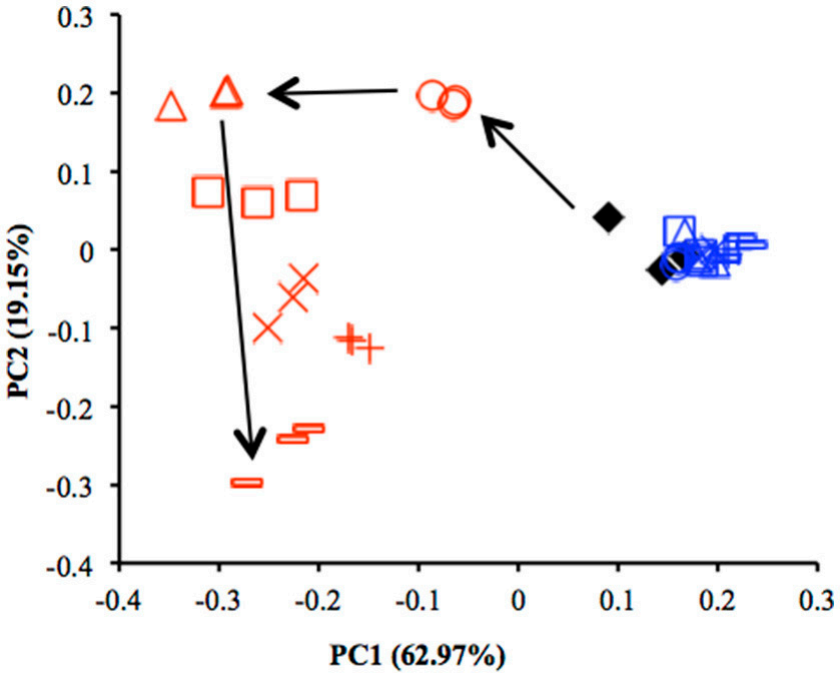
**Comparison of microbial community structures in the uppermost (0–2 mm depth, red) and deep (12–16 mm depth, blue) layers of the sediments incubated in laboratory based on principal coordinate analysis (PCoA) (***n*** = 3)**. These plots were calculated from an equal number of sequences (31 950) by weighted UniFrac analysis. ♦, before incubation (Day 0); ◦, Day 1; Δ, Day 3; □, Day 7; ×, Day 14; +, Day 28; –, Day 57. Arrows indicate the trajectory of the community structure change in the uppermost layer.

Results from qPCR showed that there was no significant difference in the copy number of 16S rRNA genes between the uppermost and deep layers (Table S4). The total number of sequences obtained from 39 sediment samples was around 2.2 million, corresponding to an average of 56,403 sequences per library. The α-diversity indices were calculated by using an equal number of sequences (31,950) subsampled 10 times from original libraries. The values were lower in the uppermost layer than in the deep layer, corresponding with the on-site survey that indicated low microbial diversity in the uppermost layer (Table S2). PCoA showed notable shifts in the uppermost-layer microbial communities (Figure 2), strongly suggesting that the exposure to the atmosphere immediately altered the physiological properties of microbes in the uppermost layer.

**Figure 3:**
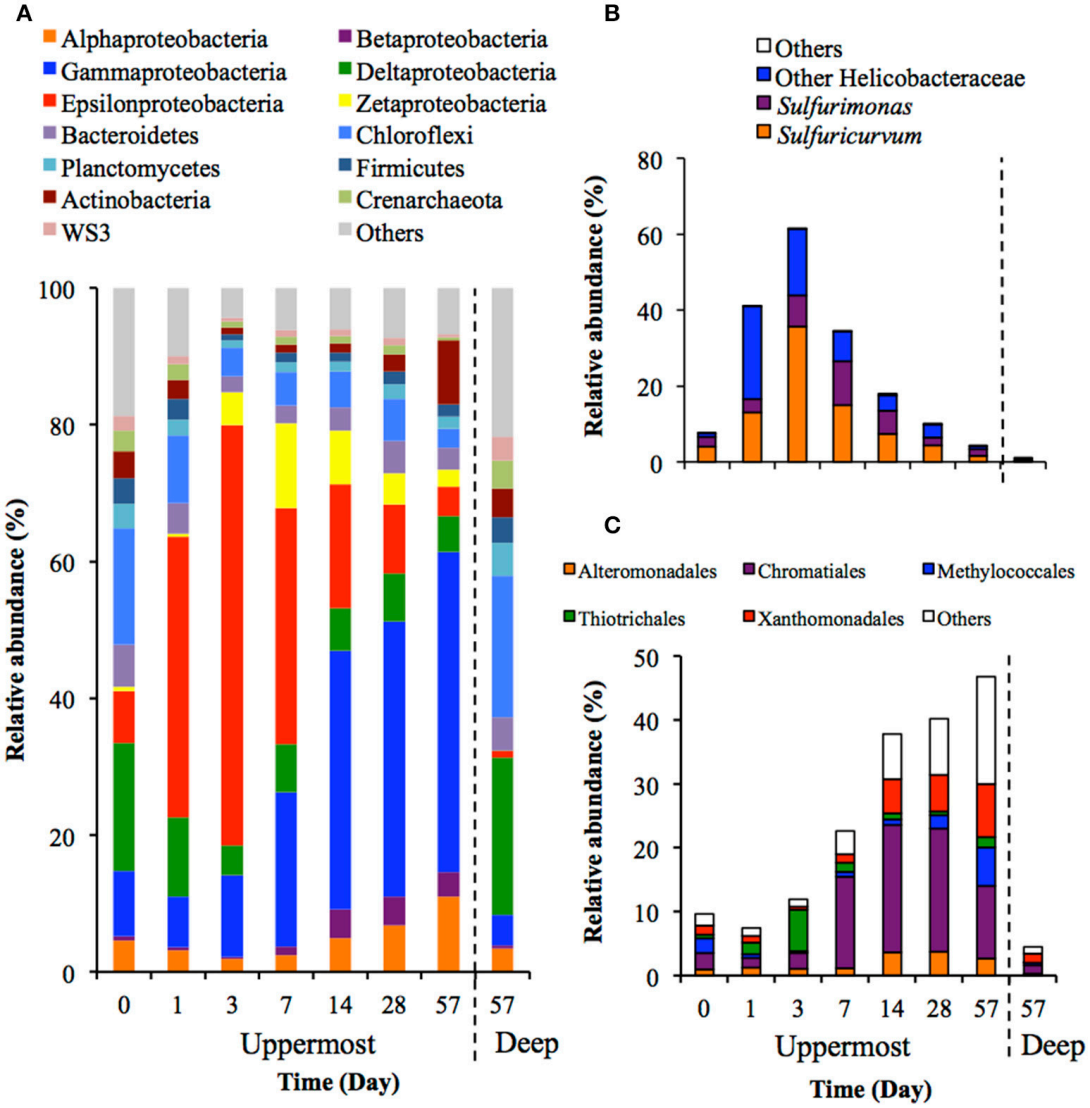
**Transition of microbial community structures in the uppermost (0–2 mm depth) and deep (12–16 mm depth) layers of the sediments incubated in laboratory (***n*** = 3)**. The bars indicate average values of three replications. Microbial communities in the deep layers (except for that at day 57) were not exhibited because the community structures were quite similar during the incubation period. **(A)** Microbial communities are categorized by phylum except for Proteobacteria that is shown by class. The fraction of the dominant phylotypes (>3% of each library) in the classes Epsilonproteobacteria **(B)** and Gammaproteobacteria **(C)** are shown in the histograms.

Figure 3 shows the succession of microbial communities of the sediment during the incubation, and the most predominant 7 OTUs in the uppermost layer at each sampling date are summarized in Table 3 and Table S5. Microbial communities in the uppermost layer changed considerably with incubation time. The relative abundance of the class Epsilonproteobacteria increased dramatically from 7.5% at day 0 to 61.5% at day 3 (Figure 3B). The family Helicobacteraceae was the most dominant taxon found in this class, and comprised the genera *Sulfuricurvum* and *Sulfurimonas* that are known as important SOB in marine sediment (Kodama and Watanabe, 2004). These dramatic succession from anaerobic chemoorganotrophic bacteria to SOB in the microbial communities strongly suggest the importance of sulfur oxidation processes in the launched marine sediment under oxic conditions. Growth of some SOB (e.g., *Sulfurovum lithotrophicum*) that did not prevail under nitrate-reducing conditions in the previous study (Aoyagi et al., 2015) was enhanced under oxic conditions in this study. The rapid proliferation of the class Epsilonproteobacteria was followed by increases in the classes Zetaproteobacteria and Betaproteobacteria (Figure 3A). Phylogenetic analysis at the OTU level showed the predominance of OTUs closely related to *Mariprofundus ferrooxydans* (EF493244) and *Gallionella* sp. (HQ117915), both of which exhibit Fe(II)-oxidizing activity (Hallbeck and Pedersen, 2005; Emerson et al., 2007). This implicates that ferrous iron oxidation occurred subsequent to, or in parallel with, the sulfur oxidation by SOB. At the end of the incubation, the class Gammaproteobacteria and the phylum Actinobacteria became dominant. Because the family Streptomycetaceae in the Actinobacteria and the orders Xanthomonadales and Methylococcales in the Gammaproteobacteria are known to exhibit chemoorganotrophy (Bowman, 2005; Saddler and Bradbury, 2005; Kämpfer, 2012), it is considered that these bacteria became metabolically active in the uppermost layer after the proliferation of chemolithotrophic bacteria (i.e., SOB and Fe(II)-oxidizing bacteria [FeOB]).

**Table 3:** **Most abundant OTUs and their closely related species found in the uppermost layer of the sediment incubated in laboratory**.

**Incubation day**	**OTU No**.	**Closest relative species^a^**	**Similarity (%)**	**Accession No**.	**Phylum/Class^b^**	**Relative abundance (%)^c^**	***p*****-value^d^**	**Putative function^e^**
1	43060	*Sulfurovum lithotrophicum*	98	CP011308	Epsilonproteobacteria	14.4 ± 0.3	<0.001^**^	SO, NR
	42344	*Sulfurimonas autotrophica*	92	CP002505	Epsilonproteobacteria	9.7 ± 0.3	<0.001^**^	Unknown
	25387	*Sulfurovum aggregans*	96	AB889689	Epsilonproteobacteria	9.2 ± 0.4	<0.001^**^	SO, NR
	49085	*Sulfurimonas* sp. 0H30-7C-S	90	AB304903	Epsilonproteobacteria	2.9 ± 0.5	0.002^**^	Unknown
	27532	*Sulfurimonas* sp. MA01	95	AB930173	Epsilonproteobacteria	1.6 ± 0.6	0.04^*^	Unknown
	49878	*Desulfobulbus elongatus*	92	CP002364	Deltaproteobacteria	1.6 ± 0.0	0.005^**^	Unknown
	21731	*Thiomicrospira psychrophila*	100	AJ404732	Gammaproteobacteria	1.2 ± 0.2	0.001^**^	SO
3	42344	*Sulfurimonas autotrophica*	92	CP002505	Epsilonproteobacteria	30.7 ± 6.4	0.001^**^	Unknown
	25387	*Sulfurovum aggregans*	96	AB889689	Epsilonproteobacteria	9.0 ± 3.4	0.01^*^	SO, NR
	43060	*Sulfurovum lithotrophicum*	98	CP011308	Epsilonproteobacteria	8.0 ± 2.1	0.005^**^	SO, NR
	49085	*Sulfurimonas* sp. 0H30-7C-S	90	AB304903	Epsilonproteobacteria	4.1 ± 0.8	0.003^**^	Unknown
	32337	*Mariprofundus ferrooxydans*	96	EF493244	Zetaproteobacteria	3.9 ± 0.2	<0.001^**^	FeO
	36501	*Thiomicrospira crunogena*	100	L40810	Gammaproteobacteria	3.7 ± 0.9	0.002^**^	SO
	30483	*Sulfurimonas denitrificans*	98	L40808	Epsilonproteobacteria	2.7 ± 1.1	0.02^*^	SO, NR
14	45161	*Thioalkalispira microaerophila*	98	AF481118	Gammaproteobacteria	18.3 ± 1.9	<0.001^**^	SO, NR
	32337	*Mariprofundus ferrooxydans*	96	EF493244	Zetaproteobacteria	4.8 ± 0.8	<0.001^**^	FeO
	6816	*Gallionella* sp. PN013	97	HQ117915	Betaproteobacteria	4.0 ± 0.2	<0.001^**^	FeO
	42344	*Sulfurimonas autotrophica*	92	CP002505	Epsilonproteobacteria	3.9 ± 0.6	0.001^**^	Unknown
	6961	*Dyella ginsengisoli*	100	KC129050	Gammaproteobacteria	3.4 ± 2.1	0.05	ChemO
	30483	*Sulfurimonas denitrificans*	98	L40808	Epsilonproteobacteria	3.1 ± 0.4	<0.001^**^	SO, NR
	16111	*Thiohalophilus thiocyanatoxydans*	96	DQ469584	Gammaproteobacteria	2.9 ± 0.5	0.001^**^	SO, NR
57	45161	*Thioalkalispira microaerophila*	98	AF481118	Gammaproteobacteria	9.0 ± 1.7	0.001^**^	SO, NR
	24485	*Streptomyces vitaminophilus*	99	AB184589	Actinobacteria	8.1 ± 2.5	0.005^**^	ChemO, NR
	908	*Salinispirillum marinum*	93	KJ195687	Gammaproteobacteria	5.9 ± 1.7	0.004^**^	Unknown
	23047	*Oleiagrimonas soli*	98	JQ658406	Gammaproteobacteria	4.8 ± 1.2	0.002^**^	ChemO, NR
	30112	*Thiohalophilus thiocyanatoxydans*	92	DQ469584	Gammaproteobacteria	3.0 ± 1.2	0.01^*^	Unknown
	45198	*Thiohalophilus thiocyanatoxydans*	96	DQ469584	Gammaproteobacteria	2.6 ± 0.7	0.003^**^	SO, NR
	6961	*Dyella ginsengisoli*	100	KC129050	Gammaproteobacteria	2.4 ± 0.4	0.001^**^	ChemO

a *The closely related species were assigned on BLAST in the DDBJ*.

b *The OTUs were characterized phylogenetically by using the QIIME software*.

c *The symbol “±” means the standard deviation of three replications*.

d p-values indicate whether the relative abundance of OTU was significantly high comparing with that in the deep layer:

* *p < 0.05*,

** *p < 0.01*.

e *The putative function of closely related species (only sequence similarities >95%). SO, sulfur oxidation; SR, sulfate reduction; FeO, Fe(II) oxidation; ChemO, chemoorganotrophy; NR, nitrate reduction*.

A variety of SOB became dominant according to the time of the incubation. Specifically, *Sulfurovum lithotrophicum* (OTU 43060), *Sulfurovum aggregans* related species (OTU 25387), *Sulfurimonas denitrificans* (OTU 30483), *Thiomicrospira psychrophila* (OTU 21731) and *Thiomicrospira crunogena* (OTU 36501) were predominant at days 1–3, whereas *Thioalkalispira microaerophila* (OTU 45161) and *Thiohalophilus thiocyanatoxydans* related species (OTUs 16111 and 45198) increased after day 14 (Table 3). The successive dominance of SOB suggests that sulfur oxidation have an advantage over chemoorganotrophy in the organic compounds- and sulfides-rich sediment during the incubation.

Microbial community structures in the deep layers during the incubation were quite similar each other, therefore, the representative data at day 57 is presented in Figure 3. The microbial communities consisted mainly of the class Deltaproteobacteria and the phylum Chloroflexi, which is consistent with the microbial communities in the deep layers of the on-site survey (Figure 1).

### Change in geochemical properties of the sediment during laboratory incubation

**Figure 4:**
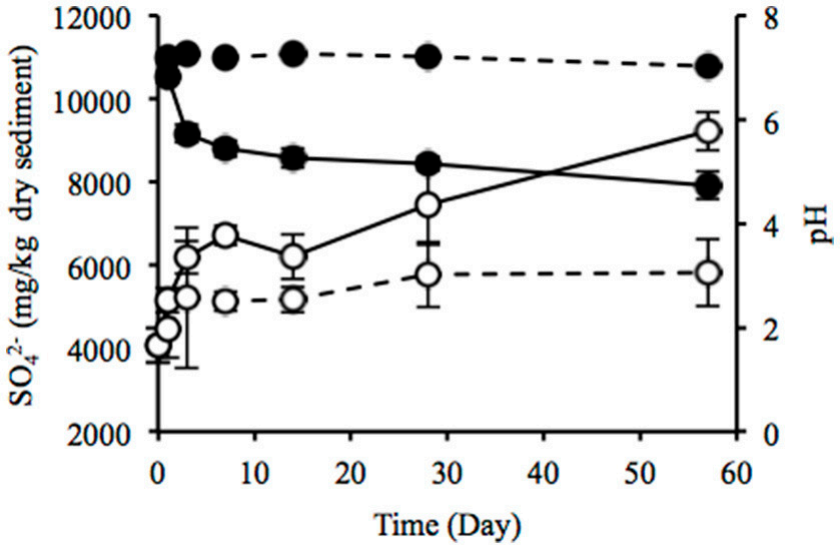
**Time-course changes in sulfate ion concentration (open circles) and pH (filled circles) of the sediments incubated in laboratory**. Solid and dotted lines indicate data in the uppermost (0–2 mm depth) and deep (12–16 mm depth) layers of the sediments. Error bars indicate standard deviations of four replications. Only sulfate ion concentrations in the uppermost layers at days 1 and 57 were conducted in triplicate.

Water content of the sediment was in the range of 55.9–58.8% during the laboratory incubation. The concentration of sulfate ion in the uppermost layer increased considerably from 4075 to 9219 mg/kg dry weight (dw), and pH decreased from 7.2 to 4.7 (Figure 4), indicating the sulfate formed by SOB acidified the sediment. Rate of the sulfate accumulation can be divided into two stages: faster rates during the first week (about 377 mg/kg dw/day) and slower rates in the succeeding period (about 50 mg/kg dw/day). In particular, the sulfate-accumulating rate in the first 3 days reached a maximum value of 705 mg/kg dw/day (i.e., 300 mg/kg ww/day). The IL in the uppermost and deep layers did not differ between the beginning and end of the incubation (Figure S5A). TC content significantly increased in the uppermost layer and the value reached 20.3 g/kg dw at the end of the incubation, whereas TN content showed no significant change (Figures S5B,C). In contrast, the DOC concentrations in both the uppermost and deep layers and the DN concentration in the uppermost layer exhibited the significant decreases (Figures S5D,E). The decreasing rate of DOC was notably higher in the uppermost layer than in the deep layer. Especially, the significant decrease in DOC in the uppermost layer was possibly due to the enhanced activities of chemoorganotrophs by the exposure to atmosphere.

## Discussion

SOB in the class Epsilonproteobacteria were predominant during the early phase of the laboratory incubation (days 1–3), whereas SOB in the class Gammaproteobacteria were predominant during the latter phase (Figure 3 and Table 3). These differences might be explained by the distinct metabolic strategies of sulfur oxidation in these SOB. The phylum Proteobacteria is known to have several pathways for sulfur oxidation. The Gammaproteobacteria has an energy-producing pathway that is kinetically advantageous if oxygen and reduced sulfur compounds are steadily supplied, while the Epsilonproteobacteria has versatile energy-producing pathways to adapt to transient environmental conditions (Yamamoto and Takai, 2011). Thus, it is plausible that the Epsilonproteobacteria dominated at the earlier stage of the incubation because of their flexibility to environmental changes.

Our previous study showed that *Sulfurimonas denitrificans* was the SOB dominated during the incubation of the launched marine sediment under nitrate-reducing conditions (Aoyagi et al., 2015). On the other hand, exposure of the sediment to the oxic conditions resulted in the proliferation of more diverse dominant SOB than those under nitrate-reducing conditions (Table 3). Sulfate accumulation rate in the previous study (1800 mg/kg wet weight (ww)/day) was almost 6 times faster than the present one (300 mg/kg ww/day), presumably due to the difference of the experimental conditions: in the previous study, sediment was suspended as slurry and anaerobically pre-incubated for 1-month before addition of nitrate. Out of the predominant OTUs found in the uppermost layer of the incubated sediment in this study, 8 OTUs (OTU 43060, 25387, 21731, 36501, 30483, 45161, 16111, and 45198) were phylogenetically related to SOB. In particular, *Sulfurimonas autotrophica, Sulfurovum lithotrophicum, Sulfurovum aggregans* and *Thiomicrospira crunogena* have been isolated from deep-sea sediments and/or hydrothermal vents (Jannasch et al., 1985; Inagaki et al., 2003, 2004; Mino et al., 2014). Other two dominant OTUs 32337 and 6816 in the uppermost layer were phylogenetically related to FeOB. The related species *M. ferrooxydans* has been isolated from hydrothermal vents and they have been known as important players in ecological iron cycling (Emerson et al., 2007; Hoshino et al., 2016). Thus, it is likely that the launched marine sediment examined harbor SOB and FeOB, both of which have been found in these aquatic ecosystems.

Dramatic environmental changes of the launched marine sediment, particularly the exposure to atmosphere, may cause strong effects geochemically and biologically. For example, the on-site sediment surface colored reddish brown (Figure S2), resulting from the formation of iron precipitates. The on-site detection of SOB and FeOB suggested that metabolic activities of these bacteria were related to the direct and/or indirect formation of iron precipitate because of the close relationship between the iron and sulfur cycling (Jørgensen and Fenchel, 1974; Jørgensen, 1977; Hsieh and Yang, 1989; Schippers and Jørgensen, 2002). Indeed, the laboratory incubation of the sediment showed the rapid increase and decrease of FeOB-related OTUs (Figure 3 and Table 3).

Heavy metals are generally preserved as metal sulfides in coastal marine sediments due to hydrogen sulfide produced by SRB (Jørgensen, 1977; Zhang et al., 2014). Relatively high metal concentrations of the launched sediment were comparable with those found in marine sediment in the Ishinomaki bay that is near the sampling site (Table 2, Imai et al., 2004). The uppermost layer exhibited apparently lower concentrations of the metals than the deep layer, which suggests that the heavy-metal mobilization was facilitated by the natural weathering of the sediment in the terrestrial environment. Both the on-site and laboratory studies demonstrated the dramatic proliferation of SOB under oxic conditions in the sediment (Figures 1, 3). The sulfur oxidation could be directly linked to the release of heavy metals from metal sulfides, as reported previously (Gadd, 2000, 2004; Sand et al., 2001; Stephens et al., 2001). Moreover, the production of sulfate from sulfur oxidation resulted in the acidification of the sediment (Figure 4). The leaching of metals at low pH has been reported previously (Evans, 1989; Masscheleyn et al., 1991; Calmano et al., 1993; Bowell, 1994). Thus, SOB might cause the mobilization of heavy metals via the direct and indirect procedures in the sediment. The time-dependent changes in sulfur compounds and heavy metals in the sediment will be necessary to clarify the involvement of SOB in these processes.

Although the launched marine sediment was rich in organic matters, chemoorganotrophic bacteria became dominant after the proliferation of chemolithotrophic SOB and FeOB (Figure 3 and Table 3). Most of the organic substances in marine sediment have been considered as being relatively persistent (Kristensen et al., 1995; Kristensen, 2000), suggesting the organic substances available for chemoorganotrophic bacteria was limited in this study. In fact, no obvious decreases in IL, TC and TN were observed during the laboratory incubation (Figures S5A–C). The increase in TC in the uppermost layer over the time course of the incubation suggests that SOB and FeOB fixed CO_2_ as a carbon source and biosynthesized organic substances (Figure S5B). SOB might facilitate the growth of chemoorganotrophic bacteria by supplementing the easily degradable organic substances for them. Because the accumulation of organic matters on the seafloor can adversely affect the ecosystem, it has long been a challenge to stimulate the degradation of organically enriched marine sediment by biological and chemical procedures (Vezzulli et al., 2004; Kunihiro et al., 2008; Wada et al., 2008; Yamamoto et al., 2008; Laverock et al., 2010).

We herein clarified the succession of microbial communities in the launched marine sediment by combining the long-term on-site survey and short-term laboratory incubation. Although the laboratory incubation was unable to recreate the on-site environment in its entirety, it provided the important information about the change of microbial communities due to the exposure to atmosphere. A variety of SOB, especially the class Epsilonproteobacteria, rapidly proliferated and induced the subsequent growth of FeOB and chemoorganotrophic bacteria. Furthermore, the metabolically activated SOB possibly contributed to the mobilization of heavy metals that bound to the sediment. Consequently, the epsilonproteobacterial SOB initiated the dynamic shift from the anaerobic to aerobic microbial communities, which play a pivotal role in element cycling in the marine sediment.

## Author contributions

HI: Main worker in this paper. TH and TA: Contribution for DNA analysis and discussion. MT: Contribution for sampling and discussion. YK: Supervisor of the first author, and contribution for discussion.

### Conflict of interest statement

The authors declare that the research was conducted in the absence of any commercial or financial relationships that could be construed as a potential conflict of interest.
