# Supplementary material for: Sulfur-Oxidizing Bacteria Mediate Microbial Community Succession and Element Cycling in Launched Marine Sediment
Source: Front Microbiol. 2017 Feb 3;8:152. doi: 10.3389/fmicb.2017.00152 (PMC5289976; doi:10.3389/fmicb.2017.00152)
Supplement: Supplementary file 1 [file DataSheet1.pdf]

Supplementary information

Sulfur-oxidizing bacteria mediate microbial community succession and element cycling in launched marine sediment

Hideyuki Ihara[1], Tomoyuki Hori[2*], Tomo Aoyagi[2], Mitsuru Takasaki[3], Yoko Katayama[4*]

1 United Graduate School of Agricultural Science, Tokyo University of Agriculture and Technology, Fuchu, Japan
2 Environmental Management Research Institute, National Institute of Advanced Industrial Science and Technology, Tsukuba, Japan
3 Department of Food and Environmental Sciences, Faculty of Science and Engineering, Ishinomaki Senshu University, Ishinomaki, Japan
4 Institute of Agriculture, Tokyo University of Agriculture and Technology, Fuchu, Japan

*** Correspondence:**

Yoko Katayama: e-mail: katayama@cc.tuat.ac.jp.

Tomoyuki Hori: e-mail: hori-tomo@aist.go.jp.

Details of prior analysis.

The launched marine sediments were collected six times (December 2011; March, July and November 2012; May and October 2013). Microbial community structures in the uppermost (0-2 mm depth) and deep (20-40 mm depth) layers of the sediments were analyzed in singlicate. Deep sequencing based on 16S rRNA genes and further analyses (i.e., phylogenetic analysis and principal coordinate analysis [PCoA]) were carried out as described in the main text. Microbial communities in the uppermost layer changed with time (Figure S3A). PCoA plot suggested that microbial communities in the uppermost layers in December 2011 and October 2013 were considerably different from those in the other dates (Figure S3B). Based on the phylogenetic information and PCoA plot, we chose the sediment samples collected in December 2011, March 2012 and October 2013 for the main triplicate analyses.

Details of metal analysis.

The on-site sediment was dried at 60 °C until the weight becomes constant, and then ground with a mortar and pestle. The resultant sediment (0.4–0.6 g) was weighed in a polytetrafluoroethylene (PTFE) beaker, added 10 mL of concentrated nitric acid and 2.5 mL of 60% perchloric acid, and then heated in the beaker covered with a PTFE watch glass at around 200 °C. As soon as white smoke was observed, the watch glass was removed and the heating was continued until the solution became syrupy. The solution was allowed to cool at room temperature and then 3.5 mL of 60% perchloric acid and 10 mL of 46% hydrofluoric acid were added. The mixture was heated without seething for around 15 min. After the solution was cooled, 10 mL of 46% hydrofluoric acid was added and the mixture was again heated until it became syrupy. After adding 5 mL of 20% hydrochloric acid and 1 mL of concentrated nitric acid, the extract was heated by covering with a watch glass for 1 h. Then, the extract was added 30 mL distilled water and heated more 1–2 h covered with the watch glass. The solution was subjected to inductively coupled plasma mass spectrometry (7500a; Agilent Technologies Inc., Tokyo, Japan) after dilution of the sample within the sensitivity range of the equipment.

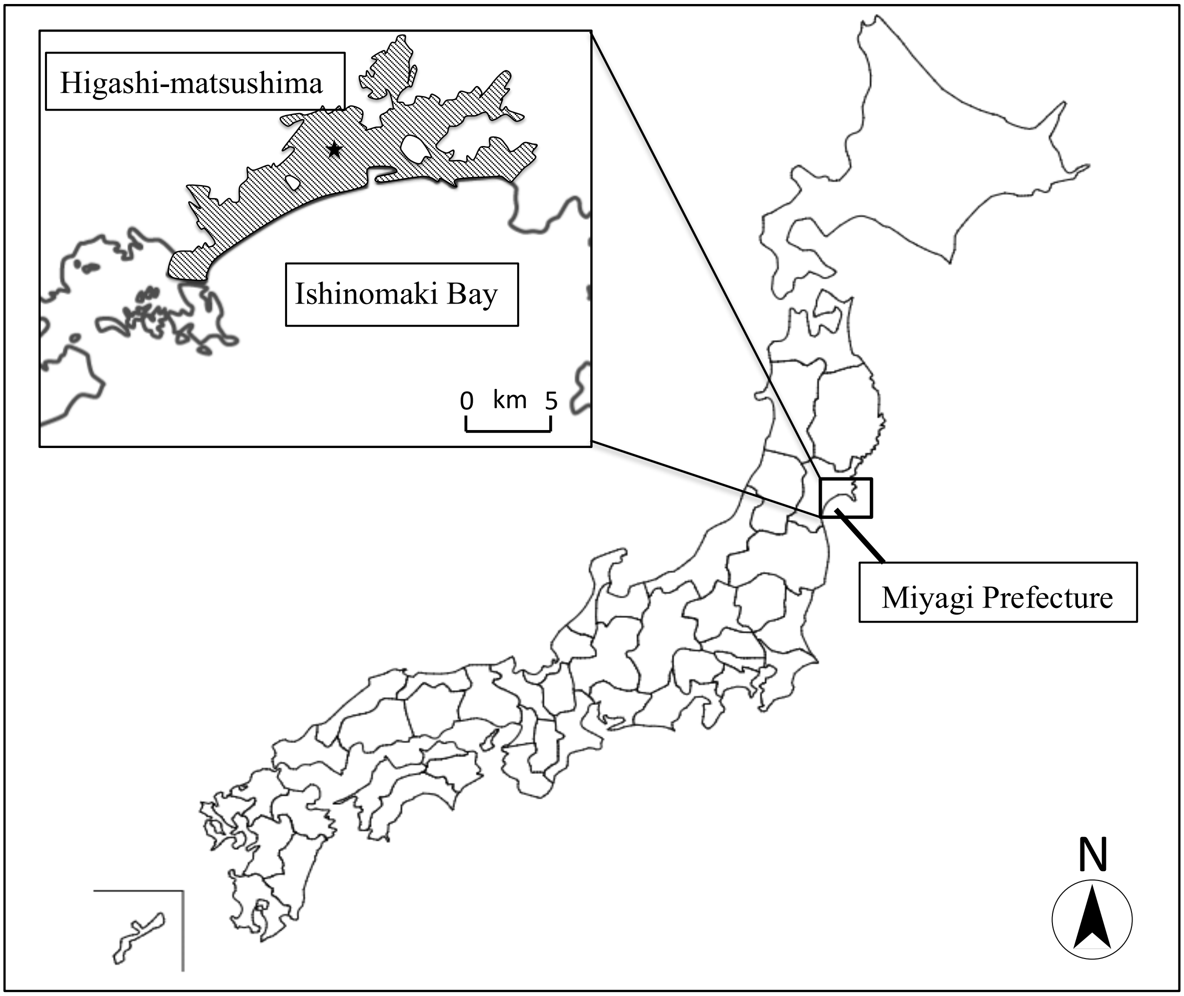

Figure S1. Location of sampling site. The launched marine sediment was collected from the area where had been used as paddy field in Higashi-matsushima city, Miyagi Prefecture, Japan. Distance of sampling site shown by the star symbol was approximately 3 km far from the shore and 1 m above the sea level. Jougawa river flows near the site. Shaded portion in inset shows the flooded area by tsunami accompanied with the Great East Japan Earthquake.

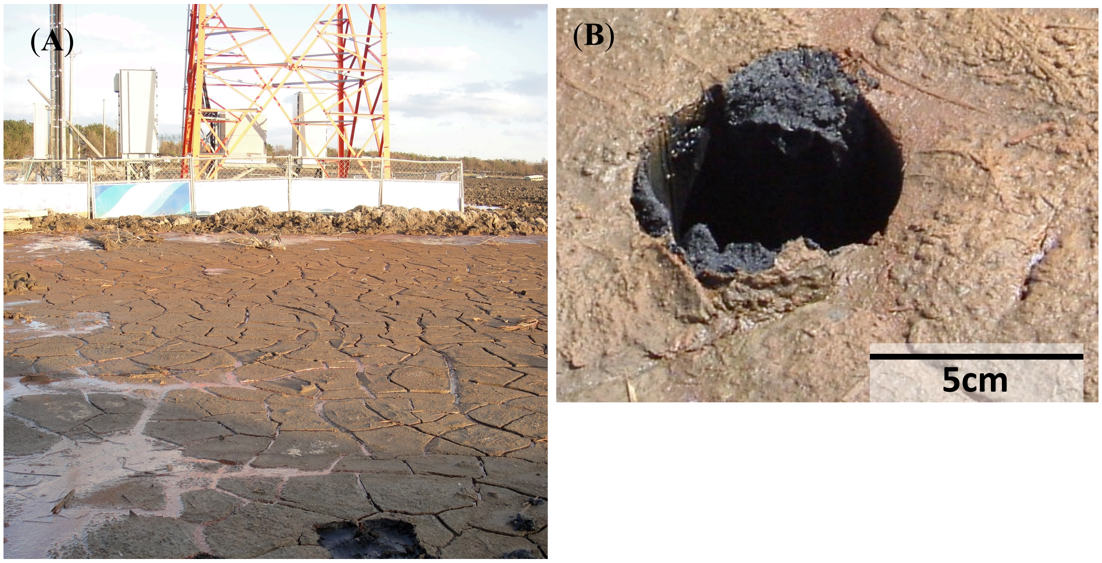

Figure S2. Photographs of the on-site sediment. (**A**) The sediment deposited on the paddy field in December 2011. Depth of the sediment was more than 30 cm. (**B**) Close-up picture of the sediment after the sampling with the core sampler. The uppermost (0-2 mm depth) layer was reddish brown while the deeper layer was black.

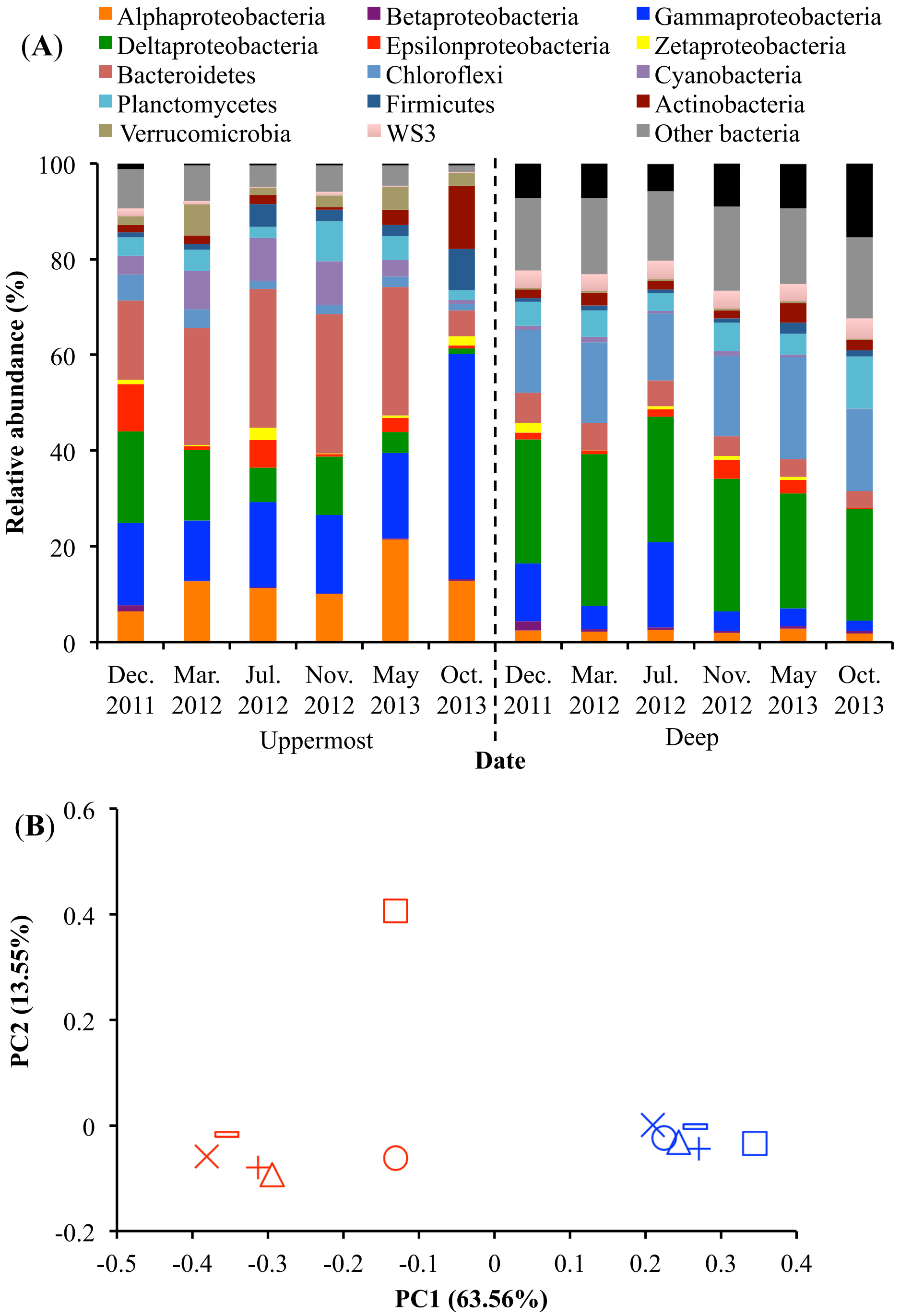

Figure S3. Prior analysis data of the on-site microbial community structures in the uppermost (0–2 mm depth) and deep (20–40 mm depth) layers by deep sequencing of 16S rRNA genes (n = 1). Sediment samples were collected in December 2011, March, July and November 2012, May and October 2013. (**A**) Microbial communities are categorized by phylum except for Proteobacteria that is shown by class. (**B**) Comparison of microbial community structures in the uppermost (0-2 mm depth, red) and deep (20-40 mm depth, blue) layers based on principal coordinate analysis (PCoA) (n=1). These plots were calculated from an equal number of sequences (15,034) by weighted UniFrac analysis. Symbols; ○, December 2011; Δ, March 2012; ×, July 2012; +, November 2012; −, May 2013; □, October 2013.

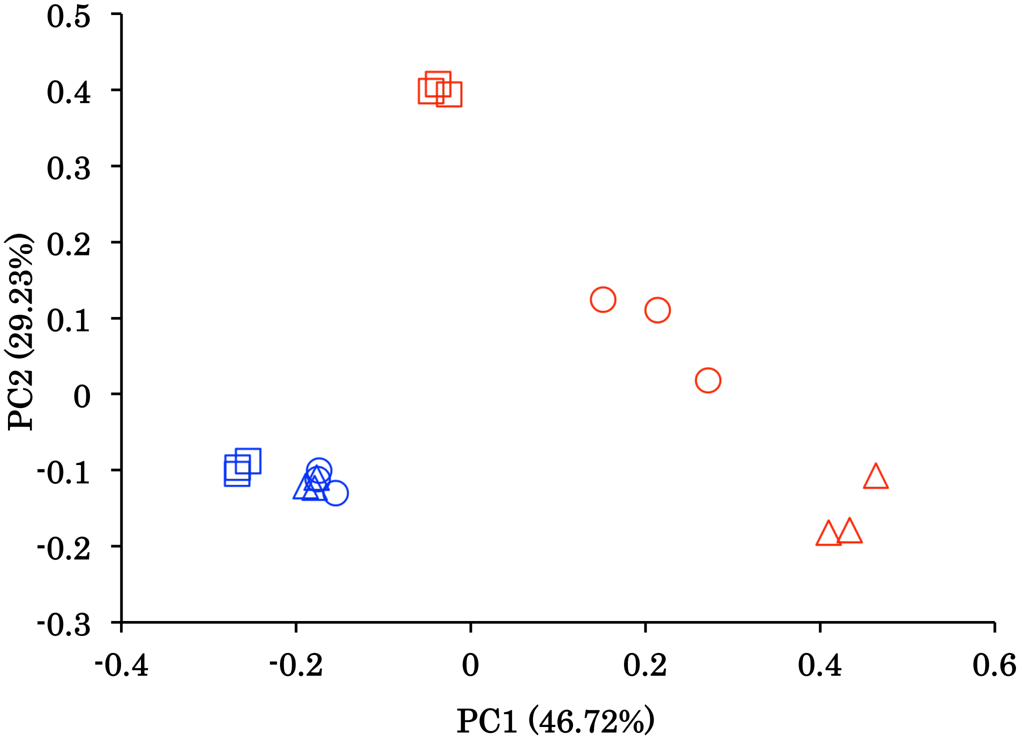

Figure S4. Comparison of the on-site microbial community structures in the uppermost (0-2 mm depth, red) and deep (20-40 mm depth, blue) layers based on principal coordinate analysis (PCoA) (n=3). These plots were calculated from an equal number of sequences (30,789) by weighted UniFrac analysis. Symbols; ○, December 2011; Δ, March 2012; □, October 2013.

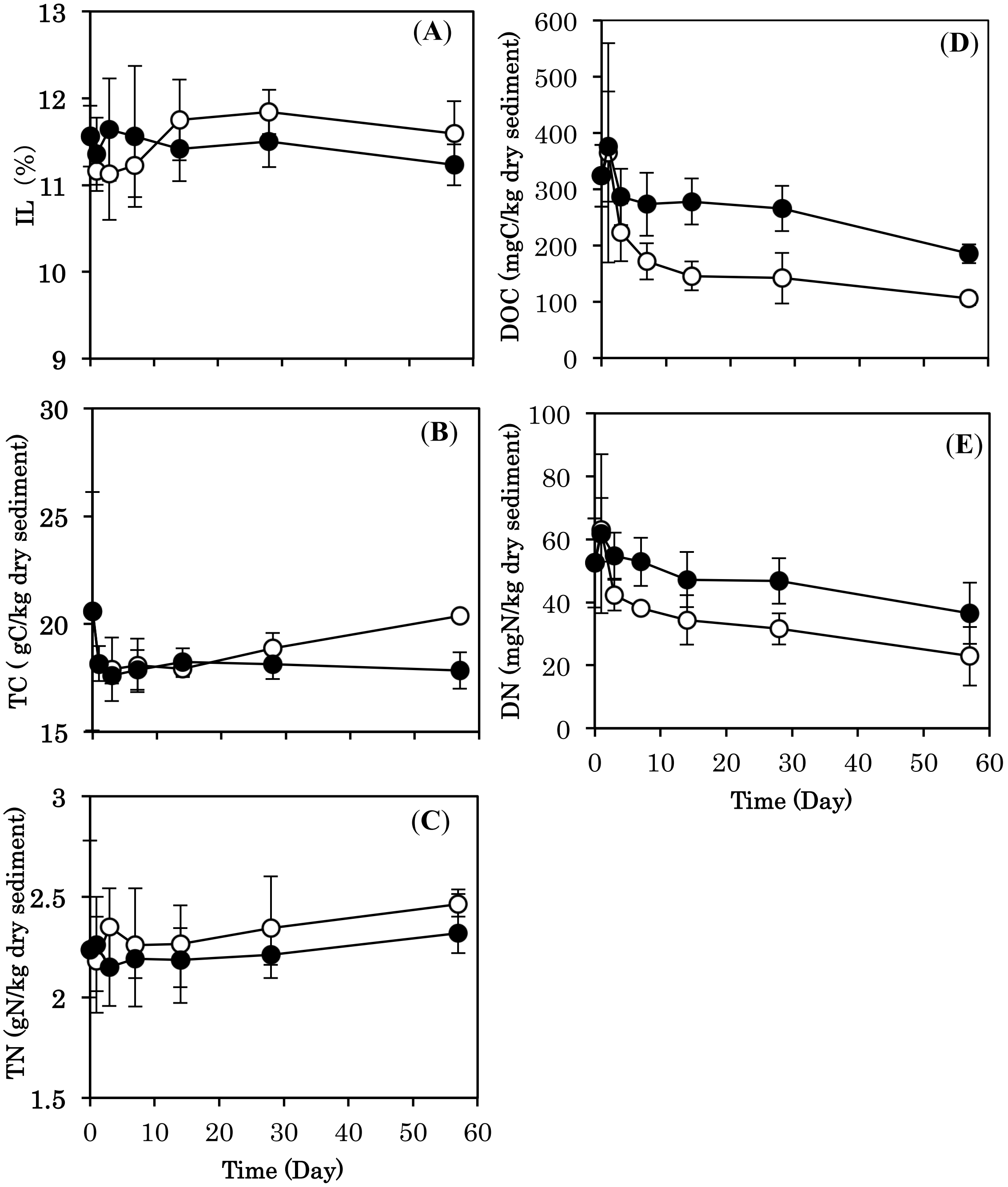

Figure S5. Time-course changes of (**A**) IL, (**B**) TC, (**C**) TN, (**D**) DOC and (**E**) DN in the sediments incubated in laboratory (n=3). Open and filled circles indicate the uppermost (0-2 mm depth) and deep (12-16 mm depth) layers, respectively. Error bars indicate standard deviations of quadruplicate samples.

Table S1 Changes of pH and sulfate concentration in the uppermost layer of on-site sediment

Sampling date	pH[a]	SO_4^{2-} (mg / kg dry sediment) [b]
Dec. 2011	4.3 ± 0.6	3317 ± 317
Mar. 2012	7.1 ± 0.2	3025 ± 95
Oct. 2013	4.4 ± 0.0	15,165

[a] The pH values were analyzed in triplicate. The symbol "±" means the standard deviation of three replications.

[b] The sulfate concentrations except for that in October 2013 were analyzed in duplicate. The symbol "±" means the variation between two replications. Due to the small quantity of obtained sample, sulfate concentration in October 2013 was measured in singlicate.

Table S2 Summary of Illumina sequencing of the on-site sediment[a] (n=3)

Layer	Sampling date	No. of sequences	No. of OTUs	α-diversity indices [b]		
				Chao1	Shannon	1/Simpson
Uppermost	Dec. 2011	38,069 ± 3753	2055 ± 82	7430 ± 773	7.4 ± 0.9	36 ± 26
	Mar. 2012	47,449 ± 4701	1838 ± 304	5546 ± 1322	8.2 ± 0.2	61 ± 13
	Oct. 2013	39,880 ± 5718	743 ± 41	2279 ± 78	6.6 ± 0.2	24 ± 5
Deep	Dec. 2011	34,808 ± 2432	2832 ± 53	14,081 ± 308	10.5 ± 0.0	310 ± 20
	Mar. 2012	40,886 ± 2216	2818 ± 30	14,036 ± 400	10.4 ± 0.1	277 ± 14
	Oct. 2013	37,556 ± 1446	2663 ± 22	13,000 ± 514	10.4 ± 0.0	289 ± 18

[a] The symbol "±" means the standard deviation of three replications.

[b] Diversity indices were calculated by using an equal number of sequences (30,789) subsampled 10 times from original libraries.

Table S3 Most abundant OTUs and their closely related species found in the uppermost and deep layers of the on-site sediment

Layer	Sampling date	OTU No.	Closely related species[a]	Similarity (%)	Accession No.	Phylum/Class[b]	Relative abundance (%)[c]	*p*-value[d]	Putative function[e]
Uppermost	Dec. 2011	1598	*Pandoraea thiooxydans*	98	EF397578	Betaproteobacteria	12.4 ± 5.5	0.02*	SO
		11292	*Sulfurimonas denitrificans*	94	L40808	Epsilonproteobacteria	8.7 ± 4.1	0.03*	Unknown
		11288	*Sulfurimonas* sp.	95	AB930173	Epsilonproteobacteria	6.5 ± 2.3	0.01**	SO
		3053	*Thiomicrospira frisia*	96	AF013974	Gammaproteobacteria	6.1 ± 2.8	0.02*	SO
		10262	*Sulfurimonas denitrificans*	98	L40808	Epsilonproteobacteria	3.5 ± 0.6	0.001**	SO, NR
	Mar. 2012	6337	*Gaetbulibacter lutimaris*	100	JF739861	Bacteroidetes	10 ± 3.0	0.005**	CO
		17127	Cyanobacterium sp.	98	FJ460075	Cyanobacteria	3.6 ± 1.2	0.008**	PS
		17093	*Luteolibacter algae*	98	AB331894	Verrucomicrobia	3.1 ± 0.9	0.004**	ChemO
		20040	*Roseovarius tolerans*	100	KP723471	Alphaproteobacteria	2.6 ± 0.2	<0.001**	ChemO
		7510	*Sagittula stellata*	100	HG315014	Alphaproteobacteria	2.3 ± 0.5	0.002**	ChemO
	Oct. 2013	17626	*Oleiagrimonas soli*	98	KM400682	Gammaproteobacteria	13.4 ± 1.4	<0.001**	ChemO, NR
		8977	*Luteibacter yeojuensis*	99	KM019785	Gammaproteobacteria	13.1 ± 3.0	0.002**	ChemO
		25201	*Dyella ginsengisoli*	99	KC129050	Gammaproteobacteria	6.1 ± 0.9	<0.001**	ChemO
		11118	*Dyella japonica*	96	KP634993	Gammaproteobacteria	4.5 ± 0.2	<0.001**	ChemO, NR
		17622	*Streptmyces hoynatensis*	96	JQ582693	Actinobacteria	2.5 ± 0.2	<0.001**	ChemO
Deep	Dec. 2011	21210	*Desulfobulbus elongatus*	92	X95180	Deltaproteobacteria	3.7 ± 0.5	-	Unknown
		14208	*Pelobacter* sp.	100	AJ271656	Deltaproteobacteria	1.8 ± 0.2	-	ChemO
		11211	*Desulfofaba fastidiosa*	95	AY268891	Deltaproteobacteria	1.5 ± 0.2	-	SR
		7299	*Thermomarinilinea lacunofontalis*	88	AB669272	Chloroflexi	1.3 ± 0.1	-	Unknown
		28763	*Desulfobulbus* sp.	96	EF442993	Deltaproteobacteria	1.3 ± 0.2	-	SR
	Mar. 2012	21210	*Desulfobulbus elongatus*	92	X95180	Deltaproteobacteria	3 ± 0.2	-	Unknown
		14208	*Pelobacter* sp.	100	AJ271656	Deltaproteobacteria	2.6 ± 0.1	-	ChemO
		7299	*Thermomarinilinea lacunofontalis*	88	AB669272	Chloroflexi	1.9 ± 0.2	-	Unknown
		7679	*Pelobacter* sp.	96	AJ271656	Deltaproteobacteria	1.8 ± 0.3	-	ChemO
		25698	*Lutimonas saemankumensis*	100	JQ661174	Bacteroidetes	1.4 ± 0.1	-	ChemO, NR
	Oct. 2013	21210	*Desulfobulbus elongatus*	92	X95180	Deltaproteobacteria	3.6 ± 0.5	-	Unknown
		7299	*Thermomarinilinea lacunofontalis*	88	AB669272	Chloroflexi	1.9 ± 0.4	-	Unknown
		30145	*Thermomarinilinea lacunofontalis*	88	AB669272	Chloroflexi	1.6 ± 0.3	-	Unknown
		6911	*Ornatilinea apprima*	90	JQ292916	Chloroflexi	1.5 ± 0.1	-	Unknown
		11211	*Desulfofaba fastidiosa*	95	AY268891	Deltaproteobacteria	1.4 ± 0.4	-	SR

[a] The closely related species were assigned on BLAST in the DDBJ.

[b] The OTUs were characterized phylogenetically by using the QIIME software.

[c] The symbol "±" means the standard deviation of three replications.

[d] *p*-values indicate whether the relative abundance of OTU was significantly high comparing with that in the deep layer: (*) $p < 0.05$, (**) $p < 0.01$.

[e] The putative function of closely related species (only sequence similarities >95%). SO, sulfur oxidation; SR, sulfate reduction; FeO, Fe(II) oxidation; ChemO, chemoorganotrophy; NR, nitrate reduction; PS, photosynthesis.

Table S4 Number of 16S rRNA gene copies and summary of Illumina sequencing of the sediment incubated in laboratory[a]

Layer	Incubation day	No. of copies (n=2)[b]	No. of sequences (n=3)	No. of OTUs (n=3)	α-diversity indices (n=3)[c]		
					Chao1	Shannon	1/Simpson
Initial[d]	-	$1.4\times10^{10} \pm 0.2\times10^{10}$	50,580 ± 4858	3196 ± 61	14,524 ± 618	10.2 ± 0.2	230 ± 57
Uppermost	1	$1.8\times10^{10} \pm 0.4\times10^{10}$	54,031 ± 9851	3016 ± 150	11,601 ± 38	8.4 ± 0.1	29 ± 0.4
	3	$2.5\times10^{10} \pm 0.2\times10^{10}$	60,909 ± 2682	2343 ± 221	7616 ± 536	5.9 ± 0.5	9.6 ± 2.6
	7	$1.7\times10^{10} \pm 0.2\times10^{10}$	59,971 ± 8183	2610 ± 355	8862 ± 1178	7.1 ± 0.5	25 ± 4.8
	14	$1.9\times10^{10} \pm 0.3\times10^{10}$	61,900 ± 6612	2667 ± 278	8832 ± 577	7.4 ± 0.3	24 ± 3.9
	28	$2.1\times10^{10} \pm 0.3\times10^{10}$	59,643 ± 5397	2855 ± 123	9693 ± 415	8.0 ± 0.3	29 ± 5.6
	57	$4.7\times10^{9} \pm 0.9\times10^{9}$	64,432 ± 3402	2286 ± 388	7049 ± 1238	7.5 ± 0.5	40 ± 7.4
Deep	1	$7.4\times10^{9} \pm 5.0\times10^{9}$	55,234 ± 1467	3368 ± 49	15,068 ± 323	10.6 ± 0.0	346 ± 14
	3	$1.2\times10^{10} \pm 0.3\times10^{10}$	52,991 ± 4773	3277 ± 42	14,553 ± 358	10.4 ± 0.1	297 ± 35
	7	$1.9\times10^{10} \pm 0.2\times10^{10}$	55,281 ± 8200	3355 ± 57	14,649 ± 197	10.5 ± 0.1	319 ± 21
	14	$1.4\times10^{10} \pm 0.1\times10^{10}$	49,383 ± 1844	3236 ± 48	14,642 ± 369	10.3 ± 0.4	231 ± 150
	28	$1.7\times10^{10} \pm 0.2\times10^{10}$	47,263 ± 14,664	3246 ± 234	14,665 ± 137	10.6 ± 0.1	354 ± 23
	57	$1.9\times10^{10} \pm 0.7\times10^{10}$	61,624 ± 13,336	3243 ± 190	14,338 ± 402	10.5 ± 0.0	304 ± 12

[a] The symbol "±" in the column of "No. of copies" means variation between two replications and the symbol in the other column means standard deviation of three replications.
[b] 16S rRNA genes per gram sediment.
[c] Diversity indexes were calculated by using an equal number of sequences (31,950) subsampled 10 times from original libraries.
[d] Sediment sample before incubation (day 0).

Table S5 Most abundant OTUs and their closely related species found in the uppermost layer of the sediment incubated in laboratory

Incubation day	OTU No.	Closely related species[a]	Similarity (%)	Accession No.	Phylum/Class[b]	Relative abundance (%)[c]	*p*-value[d]	Putative function[e]
	45161	*Thioalkalispira microaerophila*	98	AF481118	Gammaproteobacteria	12.1 ± 1.6	<0.001**	SO, NR
	32337	*Mariprofundus ferrooxydans*	96	EF493244	Zetaproteobacteria	10.7 ± 1.8	0.001**	FeO
	42344	*Sulfurimonas autotrophica*	92	CP002505	Epsilonproteobacteria	9.2 ± 0.6	<0.001**	Unknown
7	25387	*Sulfurovum aggregans*	96	AB889689	Epsilonproteobacteria	5.0 ± 1.4	0.004**	SO, NR
	46057	*Sulfurimonas sp.*	92	LC029406	Epsilonproteobacteria	4.5 ± 1.2	0.004**	Unknown
	30483	*Sulfurimonas denitrificans*	98	L40808	Epsilonproteobacteria	4.5 ± 1.9	0.02*	SO, NR
	49085	*Sulfurimonas* sp.	90	AB304903	Epsilonproteobacteria	4.3 ± 0.7	0.001**	Unknown
	45161	*Thioalkalispira microaerophila*	98	AF481118	Gammaproteobacteria	17.7 ± 1.9	<0.001**	SO, NR
	6816	*Gallionella* sp.	97	HQ117915	Betaproteobacteria	3.9 ± 1.1	0.004**	FeO
	16111	*Thiohalophilus thiocyanatoxydans*	96	DQ469584	Gammaproteobacteria	3.3 ± 0.0	<0.001**	SO, NR
28	35447	*Marinobacter guineae*	100	KX161417	Gammaproteobacteria	2.7 ± 0.3	<0.001**	ChemO, NR
	23047	*Oleiagrimonas soli*	98	JQ658406	Gammaproteobacteria	2.7 ± 0.8	0.005**	ChemO, NR
	32337	*Mariprofundus ferrooxydans*	96	EF493244	Zetaproteobacteria	2.6 ± 0.8	0.004**	FeO
	25387	*Sulfurovum aggregans*	96	AB889689	Epsilonproteobacteria	2.2 ± 0.5	0.002**	SO, NR

[a] The closely related species were assigned on BLAST in the DDBJ.
[b] The OTUs were characterized phylogenetically by using the QIIME software.
[c] The symbol "±" means standard deviation of three replications.
[d] *p*-values indicate whether the relative abundance of OTU was significantly high comparing with that in the deep layer: (*) $p < 0.05$, (**) $p < 0.01$.
[e] The putative function of closely related species (only sequence similarities >95%). SO, sulfur oxidation; SR, sulfate reduction; FeO, Fe (II) oxidation; ChemO, chemoorganotrophy; NR, nitrate reduction.
